# Isoniazid-induced alopecia

**DOI:** 10.4103/0970-2113.76304

**Published:** 2011

**Authors:** K. B. Gupta, V. Kumar, S. Vishvkarma, R. Shandily

**Affiliations:** *Department of TB & Respiratory Medicine, Pt. Bhagwat Dayal Sharma, Post Graduate Institute of Medical Sciences, Rohtak, Haryana, India*; 1 *Department of Medicine, Pt. Bhagwat Dayal Sharma, Post Graduate Institute of Medical Sciences, Rohtak, Haryana, India*

**Keywords:** Alopecia, antitubercular drugs, isoniazid

## Abstract

Isoniazid is a safe and very effective antituberculosis drug. Antimitotic agents routinely cause alopecia. Drug-induced alopecia is usually reversible upon withdrawal of the drug. Isoniazid, thiacetazone and ethionamide are the antituberculosis drugs which have been associated with alopecia. Isoniazid-induced alopecia was observed in one case and confirmed by the finding that hair growth resumed when drug removed from the regimen.

## INTRODUCTION

Isoniazid in combination with other drugs is a very effective chemotherapeutic agent in the treatment of tuberculosis. It is found to be safe, simple to administer, cost effective with high efficacy and well tolerated with fewer side effects. But sometimes it is associated with unusual side effects like polyneuropathy, hepatitis, psychosis and skin rashes.[1] Alopecia due to isoniazid has rarely been reported in literature. We describe one case complaining of hair loss with antituberculosis treatment due to isoniazid.

## CASE REPORT

**Figure 1:**
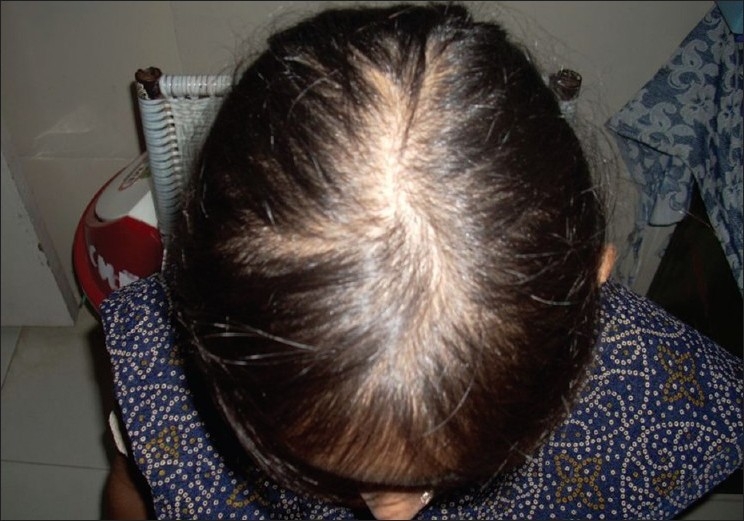
Alopecia caused by isoniazid

**Figure 2:**
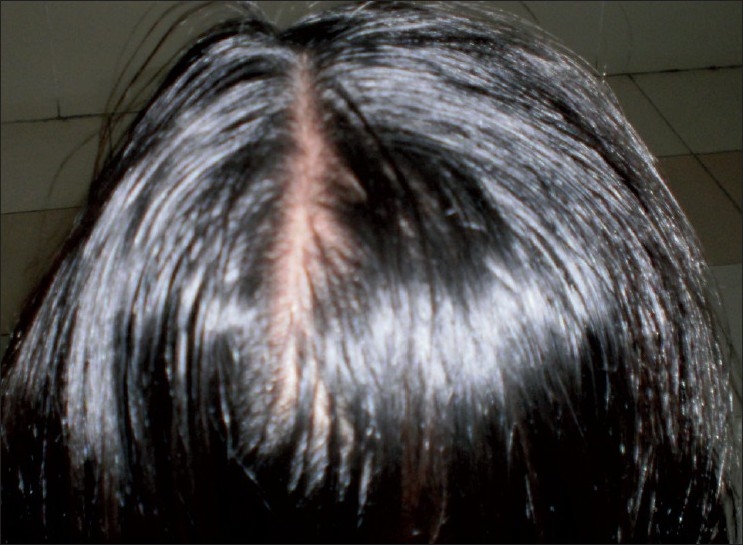
Regrowth after exclusion of isoniazid from regimen

A 30-years-old woman was put on CAT-1 DOTS containing isoniazid, rifampicin, pyrazinamide, ethambutol and vitamin B complex for pulmonary tuberculosis. After one month of start of treatment, she complained of hair fall[Figure 1]. She was HIV negative and was investigated. Isoniazid was suspected as a cause and was stopped while other drugs were continued along with vitamin B complex. After two months of stopping isoniazid, hair fall decreased and hair regrowth was observed [Figure 2].

## DISCUSSION

Alopecia may be of two types, Non-cicatricial and cicatricial. The common causes of the former are aging (pattern baldness) androgenetic alopecia (e.g. secondary to ovarian or adrenal dysfunction) traction or other trauma (trichotillomania, heat exposure) drugs, serious systemic illness, childbirth, weight loss, other stresses (telogen effluvium), skin disease (seborrheic dermatitis, eczema, tinea capitis, psoriasis, cosmetics), lupus erythematosus, hypothyroidism, hyperthyroidism, hypopituitarism, syphilis, nutritional deficiency states (kwashiorkor, marasmus, or iron, zinc, or biotin deficiency), HIV infection, congenital, hereditary. The causes of cicatricial alopecia are physical and chemical agents (cuts, burn, X-rays, radiation), infections (syphilis, leprosy, ringworm, varicella-zoster), systemic diseases like SLE, sarcoidosis, malignancy (cuteneous and others), congenital and idiopathic.[2]

Drug-induced alopecia is usually described as a diffuse non-scarring alopecia which is reversible upon withdrawal of the drug. Mainly antimitotic agents routinely cause alopecia whereas a large number of drugs may interfere with the hair cycle and produce hair loss. Drugs may affect hair follicles through two main mechanisms: (1) anagen effluvium (inducing an abrupt cessation of mitotic activity in rapidly dividing hair matrix cells) or (2) telogen effluvium (precipitating the follicles into premature rest). Anagen effluvium is a prominent adverse effect of antineoplastic agents, which cause acute damage of rapidly dividing hair matrix cells. Telogen effluvium may be a consequence of a large number of drugs including anticoagulants, vitamin A and derivatives, interferons and antihyperlipidaemic drugs. Drug-induced hair loss is usually reversible after interruption of treatment. The prevalence and severity of alopecia depend on the drug as well as on individual predisposition.[3]

Antituberculosis drugs usually cause nonscarring alopecia. Isoniazid, thiacetazone, and ethionamide have been found to be associated with alopecia. FitzGerald *et al*. reported five cases with alopecia associated with antituberculosis therapy during treatment of 141 cases of tuberculosis. Three of the five cases were HIV-positive. After about two-three months of therapy, patients complained of alopecia. Isoniazid was thought to be the cause of alopecia and supported by the finding that hair growth resumed when drug removed from the regimen.[4] Sharma *et al*. reported a case of a 32-year-old woman who developed generalized lichenoid eruptions on her body followed by diffuse loss of scalp hair of the anagen effluvium type when receiving several anti-tubercular drugs, including rifampicin, isoniazid (INH), pyrazinamide, and ethambutol for abdominal tuberculosis. INH was withdrawn; however, the other antitubercular drugs were continued. There was complete recovery of hair loss.[5] Other antituberculosis drugs have also been reported to cause alopecia which includes ethionamide and thiacetazone. Ethionamide, in combination with other anti-tuberculosis drugs, is a very effective chemo-therapeutic agent in the treatment of resistant cases of pulmonary tuberculosis. The main side-effects are gastro-intestinal, hepatitis, gynecomastia, impotence, acne, mental disturbances and convulsions.[1] Alopecia due to ethionamide is rarely reported. Arshad *et al*. reported a case of alopecia associated with ethionamide.[6] Gupta *et al*. reported diffuse toxic alopecia from thiacetazone therapy.[7] In the present case, other conditions causing alopecia are excluded by clinical examination and laboratory investigation. Growth of hair on bald areas after withdrawal of isoniazid confirmed that alopecia was due to isoniazid. The mechanism, however, is not clearly understood. The likelihood that isoniazid is responsible for alopecia is supported by the fact that hair growth resumed when the drug was omitted from the regimen. So physician should be aware of this side effect and if a patient complains of hair loss he should be reassured and difficulties with compliance need to be addressed.
